# Phage_UniR_LGBM: Phage Virion Proteins Classification with UniRep Features and LightGBM Model

**DOI:** 10.1155/2022/9470683

**Published:** 2022-04-15

**Authors:** Wenzheng Bao, Qingyu Cui, Baitong Chen, Bin Yang

**Affiliations:** ^1^Xuzhou University of Technology, Xuzhou 221018, China; ^2^University of Jinan, Jinan 250024, China; ^3^Xuzhou First People's Hospital, Xuzhou 221000, China; ^4^Zaozhuang University, Zaozhuang 277160, China

## Abstract

Phage, the most prevalent creature on the planet, serves a variety of critical roles. Phage's primary role is to facilitate gene-to-gene communication. The phage proteins can be defined as the virion proteins and the nonvirion ones. Nowadays, experimental identification is a difficult process that necessitates a significant amount of laboratory time and expense. Considering such situation, it is critical to design practical calculating techniques and develop well-performance tools. In this work, the Phage_UniR_LGBM has been proposed to classify the virion proteins. In detailed, such model utilizes the UniRep as the feature and the LightGBM algorithm as the classification model. And then, the training data train the model, and the testing data test the model with the cross-validation. The Phage_UniR_LGBM was compared with the several state-of-the-art features and classification algorithms. The performances of the Phage_UniR_LGBM are 88.51% in Sp,89.89% in Sn, 89.18% in Acc, 0.7873 in MCC, and 0.8925 in F1 score.

## 1. Introduction

Phage, which can be treated as the most abundant organism, has many important functions on earth [1, 2]. The major function of phage is to promote gene-to-gene communication [3–5]. The second function of phages is to maintain microbial diversity [6]. If the number of a bacterial species increases rapidly in a bacterial population, the corresponding bacteriophage will specifically infect this type of bacteria and kill them, so that the entire bacterial population returns to a balanced state [7, 8]. In addition, phages also participate in the Earth's material cycle [9, 10]. Blue bacteria are a kind of very important bacteria in the ocean, which can absorb carbon dioxide and convert it into glucose through photosynthesis [11]. About half of the blue bacteria will eventually be lysed by its corresponding phage and released to the entire marine environment, providing nutrients for the surrounding biological system [12–14]. Phages are also an important part of the human microbial community. Each human gut contains about 1014 bacteria, while the number of phages is 10^15-16^, 10 times more than bacteria. The function of phages is far beyond the above-mentioned issues. Nowadays, phage-targeted therapy has become a hot topic. However, phages can survive only by relying on bacterial hosts, and phages are difficult to culture. Therefore, it is particularly important to predict the interaction between phages and bacteria through bioinformatics methods [15–17].

Phage-host interaction is an effective means of studying adaptive evolution of bacteria and plays an important role in human health and disease, which may contribute to new therapeutic agents, such as phage therapy against multidrug-resistant infections [8, 18]. The continuous evolution of pathogenic bacteria and the resistance to new antibiotics may cause as many as 10 million people to lose their lives each year [19–22]. Antibiotics are usually small molecules that inhibit bacterial growth in some way. Because of their abuse and selective pressure on bacterial communities, bacteria have produced many mechanisms of resistance to these molecules over the years, such as their metabolism or excretion that render antibiotics ineffective, and the discovery of new antibiotics has become increasingly difficult.

Because bacteria are closely related to human health and the environment, ecologists and microbiologists have been studying bacterial communities to discover potential laws that are beneficial to humans and the environment. According to research, human microbial communities have been influenced by phages, and some phages will change the composition of microbial communities, resulting in changes in these communities. Phages play a key role in maintaining the microbial community structure of human and environment and provide potential tools for accurately manipulating specific microorganisms. Recent studies have further shown that interactions between phages and microbes can affect mammalian health and disease.

**Figure 1:**
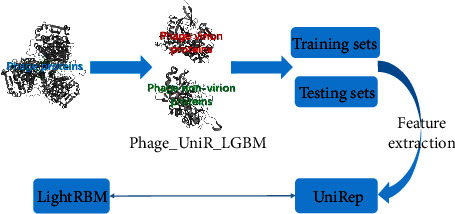
The outline of the Phage_UniR_LGBM.

It is worth noted that a great deal of features included amino acid composition [23], atomic composition (ATC) [24], chain-transition distribution (CTD) [25], pseudo amino acid composition [23], and amino acid pair [26] in the sequence level. What is more, several feature selection methods have been taken into account in this field. These approaches mainly focus on giving an increasingly effective and detailed modeling feature as the input of the classification model. On the other hand, the artificial intelligence technologies develop with the rocket speed. The machine learning algorithms, including neural network [27–29], random forest, support vector machine [30, 31], *k*-nearest neighbor [32], logistic regression [33, 34], and some deep learning models [35–37], have become one of the hottest topics among this field. In this work, we propose the Phage_UniR_LGBM. Such model utilizes the UniRep as the feature and the LightGBM algorithm as the classification model. In detailed, the dataset of phage proteins has been initially divided into 2 parts, including both the training ones and the testing ones. And then, the training data train the model, and the testing data test the model with the cross-validation. In order to demonstrate the performances of this method, the above-mentioned classification models and the features have been compared with the Phage_UniR_LGBM. We demonstrate the outline with these steps of the Phage_UniR_LGBM in Figure 1.

## 2. Methods and Materials

### 2.1. Dataset

In order to classify the phase proteins, the data employed the Ding's effort, which mainly focus on phage virion proteins researches. Such dataset is a reliable dataset, which selected with many filtering schemes [38]. Meanwhile, such dataset can hardly be treated the redundant data. The protein sequences can pairwise with any other one in low homologous. Last but not the least, several state-of-the-art methods have tested the performances with such dataset. Therefore, such dataset can be treated as a typical benchmark dataset in this field. Considering such situation, we employed such dataset as the identified data in this work. The detailed information of the employed data should be demonstrated in Table 1.

From Table 1, we can easily find that there are two types of these proteins, the 99 sequences of phage virion proteins and the 208 ones of nonphage virion proteins. The whole number of proteins is 307. According to this situation, we can define the phage virion proteins as the positive samples. So the nonphage virion proteins can be treated as the negative ones. With this information, such work can be abstracted as the typical two-type classification issue in the machine learning area. A necessary step should be taken into account. The 90% of both the positive and negative samples should be treated as the training and testing data, and the rest of the 10% of the whole dataset can be treated as the independent data in this work. It was pointed out that the training and testing dataset can hardly overlap with the independent ones. The training and testing dataset utilized the 2, 3, 4, 5, 6, 8, 10-fold cross-validation to demonstrate the stability of the Phage_UniR_LGBM.

### 2.2. LightGBM Algorithm

The gradient boosting decision tree (GBDT) [39, 40], which has the ability to learn the performances of learners, is continuously improving with several computational iterations. During the iteration of such special algorithm, several parameters of this algorithm should be listed. The current iteration of model achievement can be defined as the *F*_*c*_ (*x*). In detail, the *c* means the current iteration. With a similar theory, the *F*_*c*−*n*_ (*x*) means the last *n* iterations' model achievement, and the *F*_*c*+*n*_ (*x*) means the next *n* iterations' model ones. What is more, the loss function of the current iteration can be defined as the Loss (*y*, *F*_*c*_ (*x*)). Such algorithm can focus on searching and dropping out the weak learner *h*_*c*_ (*x*) with the minimization of the loss function in the current round. And then, the loss function's negative gradient can be calculated on the current iteration's loss function. It was pointed out that the square difference plays a significant role during this algorithm. So, such parameter can be measured with the method of the fitting *h*_*c*_ (*x*) in equation (1), and the loss function can be evaluated by equation (2).

(1) $$\begin{array}{c} h_{c}\left(x\right)=\underset{h\in H}{\arg\min}\sum L\left(y,F_{c-1}\left(x\right)+h\left(x\right)\right), \end{array}$$

(2) $$\begin{array}{c} r_{ti}=-\frac{\partial L\left(y,F_{t-1}\left(x_{i}\right)\right)}{\partial F_{t-1}\left(x_{i}\right)}. \end{array}$$

In the final step, the most potential learner can be selected with the method, shown in equation (3), in the current iteration.

(3) $$\begin{array}{c} F_{c+n}\left(x\right)=h_{2n}\left(x\right)+F_{c-n}\left(x\right). \end{array}$$

When it comes to the Light Gradient Boosting Machine, such algorithm, can be abbreviated LightGBM [41, 42], is a special type of the above-mentioned algorithm. In detail, the GBDT algorithm mainly relies on the gradient one-side sampling and exclusive feature bundling. Such two characteristics can be treated as the main contributions of effective and high-performance. However, the shortcomings can hardly be neglected that the GBDT may speed huge computation resources during the algorithm operation. So as to overcome such deficiency, the LightGBM algorithm, which has the ability to achieve the same accuracy with the 5% time-consuming, is proposed. The majority of LightGBM algorithm may follow the next four steps. Initially, the input data can be transformed with the histogram form. After such transformation, a histogram can be constructed with the same size of input integers. The constructed histogram has the ability to capture the optimal cutting point. With such approach, several unnecessary calculations can hardly be operated. So, the computation resources may save to some degree. The second step focuses on constructing a histogram's leaf nodes. With the method of a histogram for subtraction, the computational time can cut half of the traditional method. The next step utilizes the leaf-wise growth method, which is limited to the depth of tree construction. With this approach, the performances may be further accelerated than the GBDT ones. The final step of the LightGBM should not be neglected. The parallel computation can further enhance the speed without losing accuracy.

### 2.3. UniRep Feature

The features of protein description can be utilized by the UniRep [43, 44], which is a novel approach to demonstrate the protein information at various levels. With the further researches, it can be found that the amino-acid embedding approaches are learned by the UniRep. The UniRep method contains several properties in the protein level. For instance, the UniRep selected physicochemical feature with the method of amino acid residues' cluster. In detail, the whole 20 types of amino acid residues own their properties, including hydrophobic aliphatic, charged basic, charged acidic, polar neutral, unique, and hydrophobic aromatic. Such feature sets also separated protein from a huge number of structural classifications of proteins. These types can be classified with the crystallographic information. In order to evaluate the feature of identified proteins, we employ the whole protein peptide sequences from the identified phage virion proteins. So, the protein sequence can be transformed into a numeric vector. And then, we apply the LightGBM algorithm to identify the phage virion proteins numeric vectors and the nonphage ones. The input of the LightGBM is the identified numeric vector from each sample, and the output of the LightGBM is the calculated results from these samples.

### 2.4. Measurements of Performance

In this classification problem, samples can be defined as two types, including the phage virion proteins sequences and the nonphage virion proteins sequences. Defined positive samples mean the phage virion proteins sequences. On the contrary, the defined negative samples mean the nonphage virion proteins sequences. According to the definition of the classified samples, they can cause the four results in a common situation. We can easily obtain these formulations, including sensitivity, specificity, accuracy, F1 scores, and MCC. And the detailed information is shown in the following equations:

(4) $$\begin{array}{c} Sn=\frac{TP}{TP+FN}, \end{array}$$

(5) $$\begin{array}{c} Sp=\frac{TN}{TN+FP}, \end{array}$$

(6) $$\begin{array}{c} \text{Acc}=\frac{TP+TN}{TP+TN+FP+FN}, \end{array}$$

(7) $$\begin{array}{c} F1=\frac{2TP}{2TP+FN+FP}, \end{array}$$

(8) $$\begin{array}{c} MCC=\frac{TP\times TN-FP\times FN}{\sqrt{\left(TP+FP\right)\left(TP+FN\right)\left(TN+FP\right)\left(TN+FN\right)}}, \end{array}$$

where *P* is the scale of positive samples and *N* is the scale of negative ones. *T* is a set of the true predicted result, and *F* is a set of the false predicted result.

When it comes to the F1 score, such performance can be treated as an index utilized to evaluate the positive and negative samples' distribution in the field of the two-type issue. Such performance should take into account several parameters, including the four basic parameters, which are TP, FP, TN, and FN. Such performance can be treated as a harmonic average of model accuracy and recall.

Another important performance is the MCC, which is abbreviated by Matthews correlation coefficient. Such performance's value ranges from -1 to 1. It means the relationship between the outputs and computational results. Considering the true results, false-positive ones, and true-negative ones, the MCC has the ability to demonstrate the balance of the above-mentioned three parameters. The area under receiver operating characteristic, which can be shorted as the AUC, is a significant evaluation metric. Such performance shows the relationship between the label and computational result in each sample, respectively.

## 3. Results and Discussions

To understand the classification issue of the phage virion proteins sequences and the nonphage virion proteins sequences, we define the label of the phage virion protein as 1 and the label of nonphage virion protein as 0. In other words, a phage virion one is treated as a positive sample and a nonphage virion one is treated as a negative sample. Therefore, the UniRep features of each protein sequence sample as the input of the LightGBM model and the output of each sample should be compared with their own label, respectively. In order to demonstrate the stability and reliability, we utilize the 2-, 3-, 4-, 5-, 6-, 8-, and 10-fold cross-validation. After this operation, we utilize the constructed model to test the performance in the independence dataset. So, in the following part, we demonstrate the detailed processions of the Phage_UniR_LGBM.

### 3.1. Performances of Different Classification Algorithms

**Figure 2:**
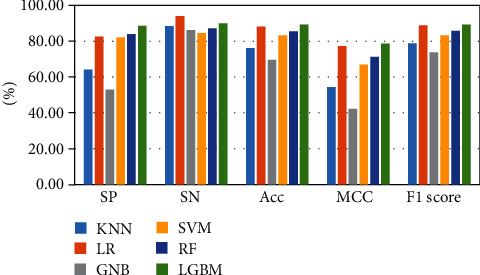
Seven classification algorithms comparisons in 2-fold validation.

**Figure 3:**
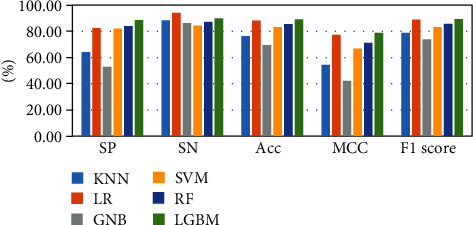
Seven classification algorithms comparisons in 3-fold validation.

**Figure 4:**
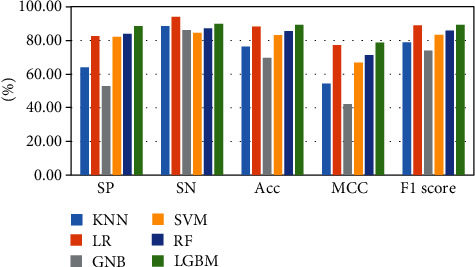
Seven classification algorithms comparisons in 4-fold validation.

**Figure 5:**
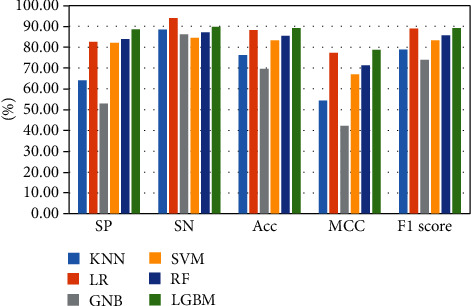
Seven classification algorithms comparisons in 5-fold validation.

**Figure 6:**
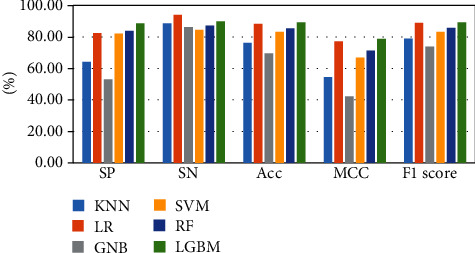
Seven classification algorithms comparisons in 6-fold validation.

**Figure 7:**
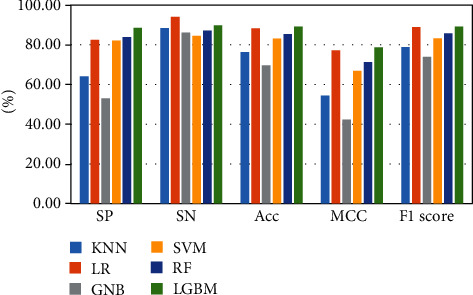
Seven classification algorithms comparisons in 8-fold validation.

**Figure 8:**
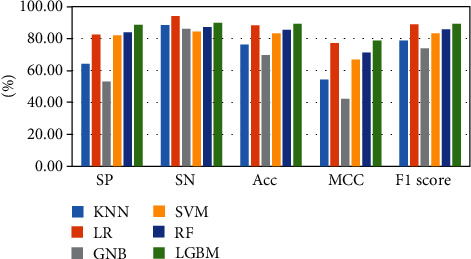
Seven classification algorithms comparisons in 10-fold validation.

The above-mentioned seven cross-validation test ways are utilized to validate the stability and reliability of the Phage_UniR_LGBM. And then, five state-of-the-art classification algorithms, including *k*-nearest neighbors, logistic regression, Gauss naive Bayes, support vector machine, and random forest, are utilized to classify the phage virion proteins and the nonphage virion proteins with the UniRep features. In order to show the stability and generality of the model, we employed the 2-fold, 3-fold, 4-fold, 5-fold, 6-fold, 8-fold and 10-fold cross validation methods.Table S1-S6 show the detailed information of Sn, Sp, Acc, MCC, and F1 scores of the LightGBM model and other state-of-the-art machine learning algorithms using various cross-validation methods. During the 5 cross-validation, we can easily find that the logistic regression algorithm can get the well performances in the Sn and the employed LightGBM model can get the well performances in the Sp. Meanwhile, the logistic regression can hardly work well in Sp. Therefore, it may cause such models own the low-performances in other three measurements, including accuracy, MCC, and F1 score. During the 2, 3, 4, 5, 6, and 8 cross-validation, the *k*-nearest neighbors model replaces the logistic regression model in Sp. It is noted that the LightGBM model's Sn is better than other compared algorithms. So, the LGBM can get the available results in the key classification performances, including Acc, MCC, and F1 score. From Figures 2–8 and Table S1-S7, we can get the conclusion that the LightGBM model can get the available effectiveness and stability during the cross-validation. On the other hand, the support vector machine and random forest model demonstrate their advantages during six parameters cross-validations.

**Figure 9:**
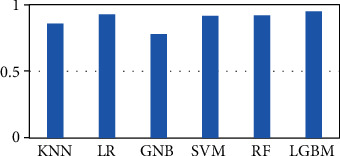
The AUCs of seven algorithms in 10-fold validation.

In order to demonstrate the performances of this imbalance classification issue, the AUC has been employed to evaluate each classification algorithm in this work. With the 2-, 3-, 4-, 5-, 6-, 8-, and 10-fold cross-validations, we find that the employed 7 cross-validations follow a similar trend in this work. So, we evaluate the AUCs of each classification algorithm, including KNN, LR, GNB, SVM, RF, and LGBM. From these results, it could be seen that LGBM has the best AUC values among seven single classifiers.  Figure 9 shows the AUC values of each algorithm in 10-fold cross-validation and the detailed values in Table S8.

### 3.2. Performances of Different Features

In this work, the UniRep features compare with several state-of-the-art features, which include amino acid composition (AAC), atomic composition (ATC), chain-transition distribution (CTD), pseudo amino acid composition (PseAAC), and amino acid pair (AAP), in the protein sequence level. To compare the performances of each feature, we utilize the above-mentioned five machine learning algorithms and the LightGBM model to test these features, respectively. With the cross-validation test, we select the 10-fold one to demonstrate their performances. And the performances of different features in these classification algorithms are shown in Tables 2– 7.

When it comes to evaluating the performances of each feature, the five employed performances should be compared, respectively. Nevertheless, the performances, such as the *Acc*, MCC, and F1 score, could be computed by the two basic performances, including the *Sp* and *Sn*. In order to more easily compare the performances of each feature, we initially compared the *Sp* and *Sn* for each one. From the performances of each feature in *kNN* model, we can easily find that the PseAAC can achieve the 66.65% in *Sp* and such performances are higher than the other five features. Meanwhile, such feature also gets the 89.51% in *Sn*, and such performance is better than other ones. It was noted that the features, including AAC, AAP, and the UniRep feature, can get the *Sn* more than 80% and the CTD, AAP, and UniRep can get the *Sp* more than 60% with the method of *kNN* classification model. Considering such phenomenon in KNN model, we propose a threshold, which is named well performance. The well performance means the evaluated performance can be higher than 70%. If the evaluated performance is 70.5%, it can be defined as the well performance. If the evaluated one is 69.99%, it can hardly be defined as the well performance. Therefore, the topic well performances are PseAAC, UniRep, and CTD in *Sp*, and the topic three well performances are AAP, UniRep, and CTD in sn with the LR classification algorithm. In the GNB model, the top three well performances are PseAAC, UniRep, and ATC in *Sp* and UniRep, PseAAC, and AAP in sn. In the SVM model, the well-performances are UniRep and PseAAC in *Sp* and PseAAC, UniRep, CTD, and AAP in *Sn*. In the RF model, the well performances are ATC, CTD, UniRep, and PseAAC in *Sp* and PseAAC, UniRep, AAP, and CTD in *Sn*. In the employed model, the well performances are UniRep and PseAAC in *Sp* and ATC, UniRep, PseAAC, and AAP in *Sn*.

After the above features comparison, we can find that the UniR_LGBM method can get the available performances than other ones. It can hardly be neglected that the PseAAC work well in several classification models.

## 4. Conclusions

In this work, a novel model, which is named Phage_UniR_LGBM, was proposed to deal with the phage virion proteins classification issue. This classification can be treated as a typical imbalance binary classification issue in the field of machine learning. In order to utilize the effective feature of the protein sequence, we employed the UniRep feature to quantitate the identified phage virion protein sequences. And then, the LightGBM algorithm was employed to evaluate the protein numeric vectors with the UniRep processing. In order to demonstrate the Phage_UniR_LGBM's stability and robustness, the 2, 3, 4, 5, 6, 8, and 10 cross-validation methods have been utilized in this work. Moreover, several typical machine learning algorithms include *k*-nearest neighbors, logistic regression, Gauss naive Bayes, support vector machine, and random forest. And then, the UniRep features were compared with several state-of-the-art features, which include amino acid composition (AAC), atomic composition (ATC), chain-transition distribution (CTD), pseudo amino acid composition (PseAAC), and amino acid pair (AAP), in the protein sequence level. From these comparisons, we find that the Phage_UniR_LGBM can be treated as an effective model to classify the phage virion protein.

From the Phage_UniR_LGBM, we find some interesting points in this classification issue. The scale of identified samples can hardly meet the need of deep learning algorithms. So, how to utilize the deep learning tools to deal with this issue? Meanwhile, the scale of negative samples and the scale of positive ones do not follow the 1 : 1 ratio. So, which strategy can be employed to deal with the typical imbalance binary classification issue in the machine learning area? The current efforts of protein sequences feature focus on the whole sequence of protein. Meanwhile, the reduction of useless feature information should be taken into account in this work. Considering such situations, we will focus on effectively solving these problems in the future efforts.

## Figures and Tables

**Table 1:** The information of phage virion proteins.

Protein type	Phage virion proteins	Nonphage virion proteins
Scale	99	208

**Table 2:** The performances of different features in KNN model.

	SP	SN	Acc	MCC	F1 score
AAC	45.50%	82.27%	63.89%	0.2986	0.6949
ATC	55.76%	75.19%	65.47%	0.3155	0.6853
CTD	63.45%	78.73%	71.09%	0.4268	0.7314
PseAAC	*66.65%*	*89.51%*	73.58%	0.4761	0.7529
AAP	62.81%	84.04%	73.42%	0.4794	0.7597
UniRep	64.09%	88.46%	76.26%	0.5440	0.7885

**Table 3:** The performances of different features in LR model.

	SP	SN	Acc	MCC	F1 score
AAC	60.23%	78.99%	69.61%	0.3993	0.7221
ATC	51.16%	76.16%	63.66%	0.2822	0.6770
CTD	66.83%	88.39%	77.61%	0.5655	0.7979
PseAAC	89.11%	82.11%	85.61%	0.7140	0.8610
AAP	56.93%	*95.91%*	76.42%	0.5738	0.8027
UniRep	82.51%	94.03%	88.23%	0.7721	0.8893

**Table 4:** The performances of different features in GNB model.

	SP	SN	Acc	MCC	F1 score
AAC	31.26%	69.80%	50.53%	0.0115	0.5852
ATC	48.22%	39.64%	43.93%	-0.1219	0.4142
CTD	41.33%	78.41%	59.87%	0.2126	0.6615
PseAAC	*69.95%*	80.89%	75.42%	0.5115	0.7400
AAP	47.16%	80.14%	63.65%	0.2892	0.6879
UniRep	52.99%	*86.17%*	69.56%	0.4225	0.7390

**Table 5:** The performances of different features in SVM model.

	SP	SN	Acc	MCC	F1 score
AAC	26.26%	54.04%	40.15%	-0.2051	0.4745
ATC	23.80%	40.53%	32.16%	-0.3618	0.3740
CTD	32.82%	79.37%	56.10%	0.1378	0.6439
PseAAC	80.42%	*84.82%*	82.62%	0.6530	0.8223
AAP	49.24%	63.33%	56.28%	0.1269	0.5916
UniRep	*82.06%*	84.44%	83.22%	0.6690	0.8327

**Table 6:** The performances of different features in RF model.

	SP	SN	Acc	MCC	F1 score
AAC	76.32%	59.27%	67.80%	0.3612	0.6479
ATC	*91.48%*	67.11%	79.30%	0.6042	0.7643
CTD	84.71%	71.47%	78.09%	0.5668	0.7654
PseAAC	78.84%	*88.49%*	83.67%	0.6765	0.8284
AAP	66.59%	72.34%	69.47%	0.3900	0.7032
UniRep	83.87%	87.16%	85.48%	0.7121	0.8572

**Table 7:** The performances of different features in LGBM model.

	SP	SN	Acc	MCC	F1 score
AAC	16.75%	48.54%	32.64%	-0.3661	0.4188
ATC	50.25%	*93.49%*	71.87%	0.4850	0.7687
CTD	48.48%	43.15%	45.81%	-0.0838	0.4433
PseAAC	84.62%	85.65%	85.14%	0.7027	0.8506
AAP	60.82%	71.91%	66.37%	0.3294	0.6813
UniRep	*88.51%*	89.89%	89.18%	0.7873	0.8925

## Data Availability

The data used to support the findings of this study are available within the manuscript and the supplementary files.

## Supplementary Materials

Supplementary Materials The file of Supplemental Data is the computational results of this classification issue. The sna means the sensitivity, the spa means the specificity, the acc1 means the accuracy, the mcca1 means the Matthews correlation coefficient, and the f1 means the f1 score in the classification issue. The other parameters are the procession parameters of this work.
