# Supplementary material for: Phage_UniR_LGBM: Phage Virion Proteins Classification with UniRep Features and LightGBM Model
Source: Comput Math Methods Med. 2022 Apr 15;2022:9470683. doi: 10.1155/2022/9470683 (PMC9033350; doi:10.1155/2022/9470683)
Supplement: Supplementary Materials — The file of Supplemental Data is the computational results of this classification issue. The sna means the sensitivity, the spa means the specificity, the acc1 means the accuracy, the mcca1 means the Matthews correlation coefficient, and the f1 means the f1 score in the classification issue. The other parameters are the procession parameters of this work. [file 9470683.f1.docx]

**Table S1 Different Classification Algorithm performances in 2-cross Validation Method.**

|  | **SP** | **SN** | **Acc** | **MCC** | **F1 score** |
| --- | --- | --- | --- | --- | --- |
| KNN | 50.63% | 82.00% | 66.32% | 0.3437 | 0.7088 |
| LR | 65.18% | 64.97% | 65.08% | 0.3015 | 0.6504 |
| GNB | 41.86% | 69.38% | 55.62% | 0.1170 | 0.6099 |
| SVM | 64.82% | 75.69% | 70.26% | 0.4076 | 0.7179 |
| RF | 66.26% | 75.06% | 70.66% | 0.4148 | 0.7190 |
| LGBM | 69.92% | 78.85% | 74.38% | 0.4896 | 0.7548 |

**Table S2 Different Classification Algorithm performances in 3-cross Validation Method.**

|  | **SP** | **SN** | **Acc** | **MCC** | **F1 score** |
| --- | --- | --- | --- | --- | --- |
| KNN | 52.55% | 84.00% | 68.28% | 0.3851 | 0.7259 |
| LR | 67.66% | 66.55% | 67.11% | 0.3421 | 0.6692 |
| GNB | 43.45% | 71.08% | 57.26% | 0.1511 | 0.6245 |
| SVM | 67.29% | 77.54% | 72.41% | 0.4506 | 0.7376 |
| RF | 68.78% | 76.89% | 72.83% | 0.4582 | 0.7389 |
| LGBM | 72.58% | 80.77% | 76.67% | 0.5352 | 0.7759 |

**Table S3 Different Classification Algorithm performances in 4-cross Validation Method.**

|  | **SP** | **SN** | **Acc** | **MCC** | **F1 score** |
| --- | --- | --- | --- | --- | --- |
| KNN | 54.48% | 87.00% | 70.74% | 0.4386 | 0.7483 |
| LR | 70.13% | 68.93% | 69.53% | 0.3907 | 0.6935 |
| GNB | 45.04% | 73.62% | 59.33% | 0.1947 | 0.6441 |
| SVM | 69.75% | 80.31% | 75.03% | 0.5034 | 0.7628 |
| RF | 71.29% | 79.64% | 75.47% | 0.5111 | 0.7645 |
| LGBM | 75.23% | 83.65% | 79.44% | 0.5909 | 0.8027 |

**Table S4 Different Classification Algorithm performances in 5-cross Validation Method.**

|  | **SP** | **SN** | **Acc** | **MCC** | **F1 score** |
| --- | --- | --- | --- | --- | --- |
| KNN | 54.48% | 87.00% | 70.74% | 0.4386 | 0.7578 |
| LR | 70.13% | 68.93% | 69.53% | 0.3907 | 0.7045 |
| GNB | 45.04% | 73.62% | 59.33% | 0.1947 | 0.6521 |
| SVM | 69.75% | 80.31% | 75.03% | 0.5034 | 0.7742 |
| RF | 71.29% | 79.64% | 75.47% | 0.5111 | 0.7761 |
| LGBM | 75.23% | 83.65% | 79.44% | 0.5909 | 0.8151 |

**Table S5 Different Classification Algorithm performances in 6-cross Validation Method.**

|  | **SP** | **SN** | **Acc** | **MCC** | **F1 score** |
| --- | --- | --- | --- | --- | --- |
| KNN | 58.32% | 92.00% | 75.16% | 0.5344 | 0.7874 |
| LR | 75.08% | 72.89% | 73.99% | 0.4799 | 0.7370 |
| GNB | 48.22% | 77.85% | 63.03% | 0.2729 | 0.6780 |
| SVM | 74.67% | 84.92% | 79.80% | 0.5991 | 0.8078 |
| RF | 76.33% | 84.22% | 80.27% | 0.6073 | 0.8102 |
| LGBM | 80.54% | 88.46% | 84.50% | 0.6922 | 0.8509 |

**Table S6 Different Classification Algorithm performances in 8-cross Validation Method.**

|  | **SP** | **SN** | **Acc** | **MCC** | **F1 score** |
| --- | --- | --- | --- | --- | --- |
| KNN | 61.53% | 95.00% | 78.26% | 0.5999 | 0.8138 |
| LR | 79.21% | 75.27% | 77.24% | 0.5452 | 0.7678 |
| GNB | 50.87% | 80.38% | 65.63% | 0.3271 | 0.7005 |
| SVM | 78.77% | 87.69% | 83.23% | 0.6673 | 0.8395 |
| RF | 80.52% | 86.96% | 83.74% | 0.6762 | 0.8425 |
| LGBM | 84.97% | 91.35% | 88.16% | 0.7647 | 0.8852 |

**Table S7 Different Classification Algorithm performances in 10-cross Validation Method.**

|  | **SP** | **SN** | **Acc** | **MCC** | **F1 score** |
| --- | --- | --- | --- | --- | --- |
| KNN | 64.09% | 88.46% | 76.26% | 0.5440 | 0.7885 |
| LR | 82.51% | 94.03% | 88.23% | 0.7721 | 0.8893 |
| GNB | 52.99% | 86.17% | 69.56% | 0.4225 | 0.7390 |
| SVM | 82.06% | 84.44% | 83.22% | 0.6690 | 0.8327 |
| RF | 83.87% | 87.16% | 85.48% | 0.7121 | 0.8572 |
| LGBM | 88.51% | 89.89% | 89.18% | 0.7873 | 0.8925 |

**Table S8 The AUCs of Seven algorithms in 10-fold Validation**

|  | **AUCs** |
| --- | --- |
| KNN | 0.8582 |
| LR | 0.9264 |
| GNB | 0.7796 |
| SVM | 0.9167 |
| RF | 0.9199 |
| LGBM | 0.9495 |
